# Bacterial Community Composition and Structure in the Littoral of Rila Mountains Glacial Lakes

**DOI:** 10.3390/life15121921

**Published:** 2025-12-15

**Authors:** Boyanka Angelova, Silvena Boteva, Ivan Traykov, Martin Tsvetkov, Anelia Kenarova

**Affiliations:** 1 Department of Ecology and Environmental Protection, Faculty of Biology, Sofia University “St. Kliment Ohridski”, 8 Dragan Tzankov Blvd., 1164 Sofia, Bulgaria; sbboteva@biofac.uni-sofia.bg (S.B.); itraykov@biofac.uni-sofia.bg (I.T.); kenarova@biofac.uni-sofia.bg (A.K.); 2 Department of Inorganic Chemistry, Faculty of Chemistry and Pharmacy, Sofia University “St. Kliment Ohridski”, 1 J. Bourchier, 1164 Sofia, Bulgaria; nhmt@chem.uni-sofia.bg

**Keywords:** cold-adapted taxa, glacial lakes, lake microbiome, metagenomics, Seven Rila Lakes, regional warming, seasonal bacterial dynamics

## Abstract

High-mountain lakes are biodiversity hotspots sensitive to increasing regional and global climate warming. However, their microbial communities remain insufficiently characterized due to their remoteness and limited accessibility. This study aimed to determine how seasonal environmental parameters shape the composition, structure and diversity of littoral bacterial communities in three glacial lakes in Rila Mountains (Bulgaria). Water samples were collected during ice-free periods in 2023 and 2024, and bacterial taxonomic composition was analysed by Next-generation sequencing. A total of 1158 bacterial OTUs were identified encompassing 18 phyla and 165 families. Actinomycetota, Pseudomonadota, and Bacteroidota were dominant at the phylum level, and Sporichthyaceae, Comamonadaceae, Chitinophagaceae and Mycobacteriaceae were most abundant among the families. Community richness and diversity peaked in June, immediately after ice melting, particularly in the highest-altitude lake (Sulzata Lake), and declined during the warm season (August), when the relative abundances of Sporichthyaceae and Mycobacteriaceae (Actinomycetota) increased. Seasonal restructuring occurred across phyla and families even in a single taxon, with water temperature and organic carbon availability identified as the main environmental drivers. The findings have improved our understanding of temperature-driven bacterial responses. They have also highlighted the vulnerability of cold-adapted taxa to regional climate warming which may contribute to more effective biodiversity conservation strategies for these unique ecosystems.

## 1. Introduction

High-mountain lakes are usually situated at elevations, often above the tree line (1800–2500 m a.s.l.), and can be found across various climatic zones. Typically formed by glacial meltwater or occupying cirques carved by glacial activity [1,2], they are important freshwater resources that host unique biological communities spanning the domains Eukarya, Archaea and Bacteria, with the latter being the dominant across the ecosystem’s ecological niches [3]. In these environments, bacteria play a crucial role in key biogeochemical processes, such as photosynthesis, organic matter degradation, nitrification and denitrification. Their ecological importance mainly stems from their contribution to the microbial loop, in which organic matter is assimilated into prokaryotic biomass, which is subsequently consumed by protists and further transferred to higher trophic levels via zooplankton predation [4]. In glacial lakes, bacteria face hostile and unfavourable conditions, such as sub-zero temperatures, long ice-covered periods, short summers, nutrient deficiencies, freeze–thaw cycles, and high UV radiation. To cope with this ecological stress, bacteria have evolved diverse adaptive mechanisms allowing them to grow and disperse successfully [5].

With the increasing recognition of bacterial importance in these systems, research has focused on better understanding of this component of the food web. In general, bacterial taxa tend to display an uneven distribution across space and time, characterised by a few dominant species that are highly abundant and many rare, niche-specialist species with specific site and seasonal preferences [6,7,8]. In this context, glacial bacterial communities are mainly dominated by several key phyla, including Actinomycetota [9,10,11,12,13], Pseudomonadota [9,10,13,14,15,16], Verrucomicrobiota [9,11,16], Bacillota [9,12], Planctomycetota [9,13,15], Bacteroidota [9,10,12,14,15,16], Cyanobacteriota [12,14,15,16] and Chloroflexota [12,15]. At the class level, Actinomycetia, along with Alpha-, Beta-, and Gammaproteobacteria, are frequently more abundant [10,16]. In lakes on the Tibetan Plateau, families such as Microbacteriaceae, Intrasporangiaceae, Isosphaeraceae, Pirellulaceae, Planctomycetaceae, Sphingobacteriaceae, Cytophagaceae, Cyclobacteriaceae, Acetobacteraceae, Rhodospirillaceae, Erythrobacteraceae, Sphingomonadaceae and Comamonadaceae have been identified [17]. Similar studies in the Pyrenees identified different families like Ilumatobacteraceae, Sporichthyaceae, Pedosphaeraceae, Chthoniobacteraceae, Chitinophagaceae, Flavobacteriaceae, Alcaligenaceae, Methylophilaceae, and Oxalobacteraceae along with Comamonadaceae [11]. Liu et al. [13] found *Sphingorhabdus*, *Rhodoferax*, and *Polaromonas* as common aquatic genera in the Tibetan glacial lakes, whereas Ilahi et al. [12] reported genera *Lactococcus*, *Pseudomonas*, *Geobacillus*, *Streptococcus*, *Caulobacter*, *Ralstonia*, *Acinetobacter* and *Bacillus* for glacial lakes of Northern Pakistan.

Bacterial diversity and community composition in glacial lakes were described in scientific literature as the outcomes of two interacting ecological mechanisms with ‘mass effect’ and ‘species sorting’ on taxa distribution [18]. The ‘mass effect’ reflects the influence of lake surroundings and the influx of allochthonous microorganisms, while ‘species sorting’ refers to the selection of the most adapted to the local environment species. Modulation of lake bacterial diversity by the ‘mass effect’ has been linked to catchment characteristics [11], altitude [19,20], land use [21], as well as climatic factors [22,23]. In contrast, in-lake conditions, such as pH, nutrients, temperature and grazing pressure, have been associated with ‘species sorting’ [20,24,25,26].

Rila is the highest mountain range on the Balkan Peninsula and the sixth-highest mountain in Europe. It hosts over 190 glacial lakes in numerous high-altitude cirque valleys. One of the most important among the lake groups is the Seven Rila Lakes (Sulzata, Okoto, Bubreka, Bliznaka, Trilistnika, Ribnoto and Dolnoto), situated in the northwestern region of Rila Mountain at an elevation between 2100 m a.s.l. and 2500 m a.s.l. Many studies have been conducted on these lakes over the years, focusing on their physical environments and biological communities of primary and secondary producers [27,28,29,30,31,32]. Additionally, bacterial communities were also analysed in terms of their abundance [33,34,35,36] and activity [37,38]. However, information on bacterial community composition and structure, as well as bacterial environmental dynamics and habitat preferences, remains limited.

To address these knowledge gaps, this study employed 16S rRNA gene sequencing in order to characterise the littoral bacterial communities of the Sulzata, Okoto, and Bubreka lakes across four ice-free sampling campaigns conducted in 2023 and 2024. Particularly, the study aimed to (i) determine the taxonomic composition, structure and diversity of bacterial communities; and (ii) assess how seasonal variations in the lakes’ water temperature and physicochemical parameters influence bacterial community structure. We hypothesised that: (i) bacterial communities would comprise taxa commonly reported from other glacial lakes, along with site- and season-specific lineages; and (ii) water temperature would be the primary driver of seasonal shifts in community structure, with the magnitude of these changes reflecting the variability in environmental conditions. Our findings will contribute to a clearer understanding of how ongoing climate warming threatens high-mountain freshwater ecosystems, as rising temperatures are expected to alter microbial community structure and potentially lead to the loss of cold-adapted taxa.

## 2. Materials and Methods

### 2.1. Study Sites and Sampling Procedure

**Figure 1:**
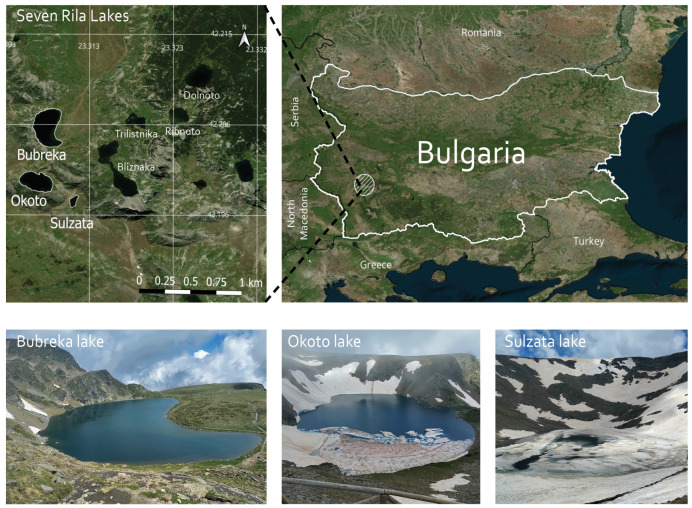
Map of the Seven Rila Lakes cirque, northwest Rila Mountains, Bulgaria with the highest situated Sulzata (42°11′50.0′′ N 23°18′38.9′ E), Okoto (42°11′57.5′′ N 23°18′23.1′ E) and Bubreka (42°12′19.6′′ N 23°18′26.4′′ E) lakes. The map was created in QGIS v. 3.40 [39].

The studied lakes are the highest situated in the cirque of the Seven Rila Lakes (Figure 1). Sulzata (Sul) Lake is the smallest of the three, situated at 2535 m a.s.l. with an area of 0.7 ha and a maximum depth of 4.5 m. Okoto (Oko) and Bubreka (Bub) lakes are situated at 2440 m a.s.l. and 2282 m a.s.l., respectively, with areas of 6.8 ha and 8.5 ha, and max depths of 37.7 m and 28.0 m. Oko is the deepest cirque lake in Bulgaria. The three lakes are not interconnected and each of them drains into the lower-situated Bliznaka Lake, which is outside the scope of this study.

Sampling campaigns were conducted in the ice-free periods of October 2023 and June, August, and October 2024. For molecular and physicochemical analyses, water was taken from the whole depth of the littoral zone at three points per lake. All samples were collected in sterile containers and kept at 4 °C during transportation to the laboratory.

### 2.2. Environmental Parameters

Dissolved oxygen (DO; mg/L), water temperature (T; °C), electrical conductivity (EC; μS/cm), and pH were measured in situ in lake littorals (10–20 cm depth) using handheld meters WTW (Oberbayern, Germany) and Hanna (Salaj, Romania). Physicochemical parameters measured in the laboratory were conducted within 48 h of sampling, and the data are presented as mean values (±standard deviation) for each lake and sampling month. The dissolved carbon (DC; mg/L) and dissolved inorganic carbon (DIC; mg/L) were analysed by high-temperature (850 °C) catalytic oxidation using Elementar Vario Select TOC analyser (Langenselbold, Germany). Dissolved organic carbon (DOC; mg/L) was calculated as the difference between DC and DIC. The dissolved organic nitrogen (DON; mg/L) was measured during the combustion process by an electrochemical cell connected to the TOC analyser. Each sample was measured in triplicate, with a standard deviation of less than 1%. Prior to the analysis, the samples were filtered through glass fibre filters (Whatman GF/F; 0.7 μm, Buckinghamshire, United Kingdom). Unfiltered samples were used to measure total nitrogen (TN; mg/L) [40] and total phosphorus (TP; mg/L) [41]. Chlorophyll-a (Chl-a; μg/L) concentration was determined according to [42]. All colorimetric analyses were performed using a CECIL CE 3021 spectrophotometer (Cambridge, UK).

DOC aromaticity [43] was calculated according to Equation (1):

(1) SUVA254=A254DOC

where: A is the absorbance at 254 nm measured in inverse meters (m^−1^) divided by the DOC concentration measured in milligrams per litre (mg/L).

The ratio DOC/DON shows the origin of organic carbon and was calculated according to [44]. DOC/DON ≤ 10: algal origin; DOC/DON > 20: terrestrial origin, and 12 < DOC/DON < 17: multiple DOC sources.

### 2.3. Molecular Analysis

A single composite sample of 1500 mL for each lake and sampling campaign was generated by mixing three 500 mL aliquots from the littoral (12 representative samples; 3 lakes × 4 sampling months) for capturing the heterogeneity of the bacterial communities. Water samples were filtered through 0.22-µm nitrocellulose membranes (47 mm diameter; GVS, Sanford, FL, USA) within 24 h. Filters were stored at −20 °C for subsequent DNA extraction.

#### 2.3.1. DNA Extraction

Total DNA was extracted from the 12 filtered water samples using the Quick-DNA™ Fecal/Soil Microbe MiniPrep Kit (Zymo Research Corp., Irvine, CA, USA), following the manufacturer’s protocol. DNA concentration and purity (A260/280 ratio) were assessed using a micro-volume UV/Vis spectrophotometer (Biobase BK-CW500, Jinan, China), and DNA integrity was evaluated by electrophoresis on a 1% agarose gel. The eluted DNA samples were stored at −20 °C until they were shipped and sequenced.

#### 2.3.2. Next-Generation Sequencing (NGS)

Next-generation sequencing (NGS) of the extracted DNA was carried out by Novogene Co. Ltd. (Cambridge, UK) using the Illumina HiSeq platform, which generates approximately 30,000 tags per sample. For bacterial identification, the V3–V4 hypervariable region of the 16S rRNA gene was amplified using the primer pair 341F/806R. Primary bioinformatic analyses were also conducted by Novogene Co., Ltd. (UK).

#### 2.3.3. Data Processing and Analysis of Operational Taxonomic Units (OTUs)

Raw sequencing data generated by the NGS platform were initially demultiplexed and quality-trimmed. Paired-end reads were merged using FLASH software (v. 1.2.7) [45]. Quality filtering on the raw tags was performed using the FASTP (v. 0.23.1) software to obtain high-quality clean tags following the criteria described by [46,47]. The tags were compared with the reference SILVA database (16S/18S) to detect chimera sequences. The effective tags were obtained by removing the chimera sequences with the VSEARCH package (v. 2.16.0) [48]. OTU clustering at ≥97% similarity was carried out using UPRASE (v. 7.0.1001) [49]. Representative sequences for each OTU were taxonomically assigned by comparison with the SILVA SSU rRNA database (v. 138) using Mothur software (v. 1.48.0) [50,51]. All taxonomic names are based on The List of Prokaryotic names with Standing in Nomenclature (LPSN) [52]. Sequences identified as mitochondria, chloroplast, archaea or unidentified bacteria were removed from the datasets. The sequencing data have been deposited in the NCBI Sequence Read Archive (SRA) under the BioProject accession number PRJNA1291052.

### 2.4. Statistical Analysis

Alpha diversity was estimated using the Shannon–Wiener and Simpson diversity indices [53], as well as the richness indices Chao1 [54], and ACE [55,56]. Venn diagrams representing shared and unique OTUs were generated using the “VennDiagram” package in R software (v 4.0.3).

Based on the idea that bacterial response is driven by environmental changes, successive k-means clustering was performed according to water temperature, starting with k = 2, then the means of measured water parameters were calculated for each cluster. To assess between-cluster differences in lake environments, an ANOVA was conducted, followed by post hoc Tukey’s test. Clusters were named ‘seasons’ without the term corresponding to the meteorological definition for season. SIMPER analysis was conducted to evaluate dissimilarity between these seasons and identify the main abiotic factors contributing to the differences. Redundancy Analysis (RDA) was applied to assess the taxonomic similarity among bacterial communities at family level, and to explore relationships between environmental variables and community composition. Pearson correlation analysis was done in order to examine linear relationships between water parameters. All statistics were performed using R (v. 4.03) [57] and PAST (v. 4.03) [58] software packages at a significance level of *p* < 0.05.

## 3. Results

### 3.1. Environmental Properties

Several key water parameters were measured during the study period, revealing clear altitudinal and seasonal patterns (Table S1). Higher-elevation lakes (Sul and Oko) remained consistently colder, whereas Bub Lake was the warmest during the ice-free period. All lakes were generally well oxygenated and near neutral in pH. High TN/TP ratios and low Chl-a concentrations indicated persistent phosphorus limitation of primary production. DOC concentrations varied widely, reflecting fluctuations in both autochthonous and allochthonous inputs.

Pearson correlations (Table S2) identified several ecologically meaningful relationships—higher water temperatures were associated with: lower DO and reduced DOC and DC, underscoring temperature-driven effects on oxygen dynamics and organic carbon turnover. Contrasting correlations between Chl-a and nutrients further highlighted the dominant role of phosphorus in regulating primary production. Positive associations between DON and carbon sources indicated tight coupling between nitrogen and carbon cycling.

K-means clustering of water temperature produced an optimal partition of lake physical environments into three clusters (seasons)—‘cold’, ‘transition’, and ‘warm’ with samples from different lakes and years grouped together (Table 1). The ‘cold’ cluster contained environments from October 2023 (Sul) and 2024 (all lakes), as well as June 2024 (Sul and Oko). The ‘transition’ cluster included October 2023 (Oko and Bub) and June 2024 (Bub). All August 2024 environments belonged to the ‘warm’ cluster. Differences in mean water temperature among clusters were significant (*p* < 0.05).

SIMPER analysis (Table S3) revealed 57% overall average dissimilarity among seasonal clusters, with the primary contributors to variability being both the quality (DC, DOC and DIC) and quantity (DOC/DON; SUVA_254_) of lake carbon.

### 3.2. Bacterial Alpha Diversity

The Alpha diversity of the amplicon sequence data was evaluated using the OTUs’ abundance, as well as richness (Chao1 and ACE) and diversity (Shannon and Simpson) indices (Table 2).

The Coverage index, reflecting sampling depth, exceeded 95.0% for all samples, indicating that sequencing depth was sufficient to reliably characterise the bacterial community composition in the study. The alpha-diversity indices indirectly reflected the community heterogeneity across lakes and sampling months. Generally, the Chao1 and ACE richness estimators were higher than the observed OTUs, likely due to the inclusion of singleton species. This overestimation may also stem from PCR-related artifacts (e.g., chimeras and mutations) and sequencing errors, which can lead to detection of the so-called ‘unusual species’ [12,59,60]. However, the Shannon/Simpson diversity indexes, which are less affected by the PCR and sequencing-based errors showed a similar pattern of changes over time, effectively overcoming the bias associated with richness estimates. Overall, the indices showed the highest mean values in June 2024, and the highest richness in Sul Lake and diversity in Bub Lake. Pearson correlation analysis revealed significant negative relationships between water temperature and bacterial richness (r = −0.49; *p* = 0.015) as well as Shannon diversity (r = −0.63; *p* = 0.001).

When calculated alpha diversity referring to abiotic seasonal clusters, a significant between-cluster difference in bacterial diversity (F ≥ 5.34, *p* ≤ 0.003) was observed, in contrast to its insignificance for OTU abundance (F ≤ 3.16, *p* ≥ 0.09) and OTU richness (F ≤ 2.47, *p* ≥ 0.21).

**Figure 2:**
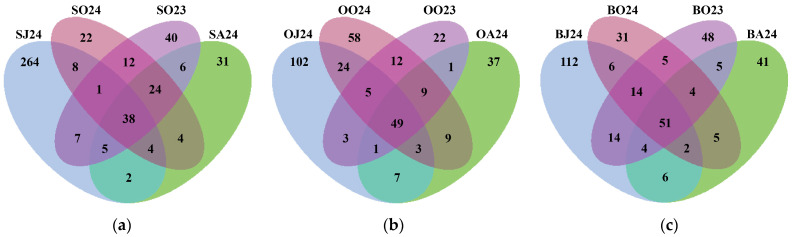
Venn diagram of species-specific OTUs distribution in water samples of (**a**) Sul Lake (S), (**b**) Okoto Lake (O) and (**c**) Bub Lake (B) observed in June 2024 (J24), August 2024 (A24), and October 2023 (O23) and 2024 (O24).

Next-generation sequencing of metagenomic DNA from the 12 water samples contained a total of 1158 OTUs. Sul Lake had the highest OTU richness with 468 OTUs (40.41%), followed by Bub Lake with 348 OTUs (30.05%) and Oko Lake with 342 OTUs (29.53%). The number of seasonally shared bacterial OTUs was 51 (14.66%) for Bub Lake, 49 (14.33%) for Oko Lake, and 38 (8.12%) for Sul Lake (Figure 2), suggesting greater seasonal shifts in bacterial communities in Sul Lake compared to the other lakes. The highest number of unshared OTUs across all lakes was observed in June 2024, suggesting highest between-lakes beta diversity. The shared OTUs between October 2023 and October 2024 (75 for each Sul and Oko lakes, and 74 for Bub Lake) demonstrated the existence of a stable month-specific fraction of microbiome.

### 3.3. Bacterial Community Composition on a Phylum and Family Levels

#### 3.3.1. Bacterial Composition at a Phylum Level

A total of 18 bacterial phyla were identified, with the highest richness detected during the ‘cold’ season (20 phyla), followed by the ‘transition’ (15) and ‘warm’ (10) seasons. Overall, 806 OTUs were classified within these phyla, including 269 OTUs assigned to Pseudomonadota, 155 to Actinomycetota, and 138 to Bacteroidota. Other phyla with relatively high OTUs richness included Bacillota (48 OTUs), the CPR group (40 OTUs), Verrucomicrobiota (37 OTUs), Chloroflexota (25 OTUs), Cyanobacteriota (20 OTUs), Acidobacteriota (17 OTUs), and Armatimonadota (13 OTUs). Minor phyla were represented by only 1 to 8 OTUs each. Within Pseudomonadota, OTUs were distributed among the classes Alphaproteobacteria (117 OTUs), Betaproteobacteria (105 OTUs), and Gammaproteobacteria (47 OTUs).

**Figure 3:**
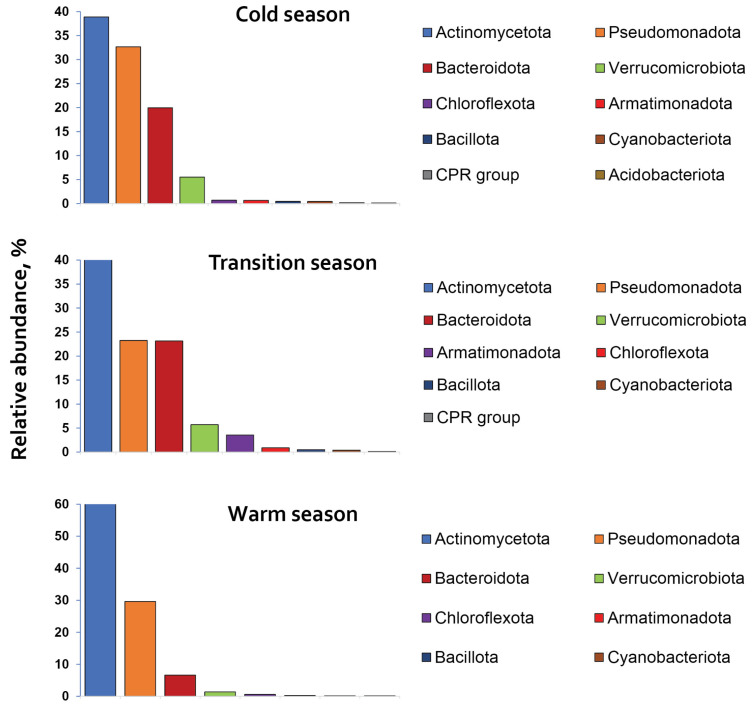
The relative abundance of bacterial phyla, represented over 0.1% of the total, distributed across the ‘cold’, ‘transition’ and ‘warm’ seasons in the littoral waters of Rila lakes.

The relative abundances of bacterial phyla varied across seasonal clusters, with their seasonal profiles presented in Figure 3.

Actinomycetota, Pseudomonadota, and Bacteroidota were identified as core phyla with seasonal-round distribution and relative abundances on average of 47.23%, 28.50%, and 16.57%, respectively. Verrucomicrobiota (4.2%) and Armatimonadota (3.5% during the ‘transition‘ season) were also among the most abundant phyla. All remaining phyla, except the above-mentioned, were identified as minor taxa, each comprising less than 1% of the total relative abundance. Actinomycetota and Pseudomonadota accounted together from 65% (‘transition’ season) to 91% (‘warm’ season) of the total bacterial abundance during the seasons.

Seasonal variability in bacterial communities was observed (Figure 3). The relative abundances of Actinomycetota and Verrucomicrobiota increased from ‘cold-transition’ to ‘warm’ season, in contrast to Bacteroidota, which decreased in that direction. Pseudomonadota maintained relatively stable levels during the seasons, ranging from 23.2% (‘transition’) to 32.6% (‘cold’).

Seasonal shifts in phylum relative abundances and OTU richness showed significant positive correlations for Bacteroidota, Verrucomicrobiota and Armatimonadota (r ≥ 0.80; *p* ≤ 0.002), and a strong negative correlation for Actinomycetota (r = −0.99; *p* < 0.0001). In contrast, Pseudomonadota relative abundance had a weak and statistically insignificant correlation with OTU richness (r = 0.28; *p* = 0.88). In the most abundant phyla Actinomycetota and Pseudomonadota, OTU richness peaked in June 2024, forming an altitudinal gradient: for Pseudomonadota, 119 (Sul)–80 (Oko)–79 (Bub); and for Actinomycetota, 68 (Sul)–63 (Oko)–61 (Bub). Moreover, the shift in Actinomycetota relative abundance was also influenced by the rate of lake warming. Its relative abundance increased in ‘warm’ compared to the ‘cold’ season from 38% to 72% in the total bacterial abundance for Sul Lake, from 34% to 59% for Oko Lake, and from 50% to 51% for Bub Lake.

#### 3.3.2. Bacterial Composition at Family and Genus Levels

**Figure 4:**
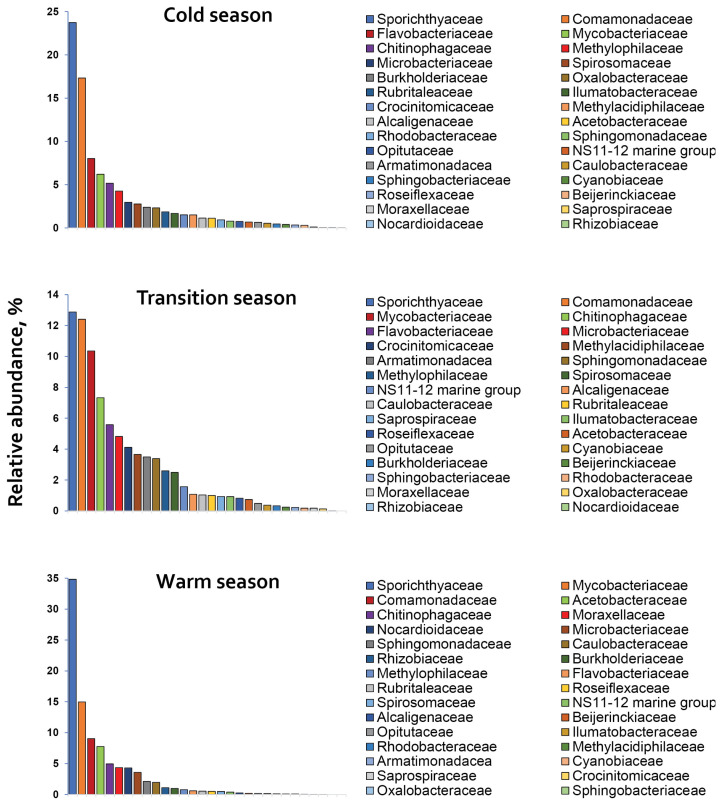
The relative abundance of the top 30 bacterial families distributed across the ‘cold’, ‘transition’ and ‘warm’ seasons in Rila lakes’ littoral waters.

Further analysis of the microbial communities identified 165 bacterial families (Table S4). The most OTU abundant families were Comamonadaceae, Chitinophagaceae, Acetobacteraceae, Flavobacteriaceae, Sphingomonadaceae, Sporichthyaceae, and Sphingobacteriaceae. Among these, Sporichthyaceae (23.81 ± 10.9%), Comamonadaceae (12.92 ± 4.2%) and Chitinophagaceae (5.82 ± 1.3%) along with Mycobacteriaceae (10.51 ± 4.4%), dominated bacterial communities) during the overall sampling period (Figure 4). Acetobacteraceae (7.77%) and Flavobacteriaceae (6.80 ± 1.7%) were also dominant in the ‘warm’ and in the ‘cold’ season, respectively. Sphingomonadaceae accounted for between 0.78% and 3.38%, while Sphingobacteriaceae represented less than 1.0% of the bacterial abundances during the overall period of study.

As a general pattern observed among the bacterial families in the Rila Lakes, most were represented by several genera, although typically only one or a few dominated the respective communities. For instance, the family Sporichthyaceae was dominated by the *hgcI* clade, Comamonadaceae by the genus *Limnohabitans* and Chitinophagaceae by the genus *Ferruginibacter*. Other families, such as Flavobacteriaceae and Mycobacteriaceae, were represented by a single genus—*Flavobacterium* and *Mycobacterium*, respectively (Table 3).

**Figure 5:**
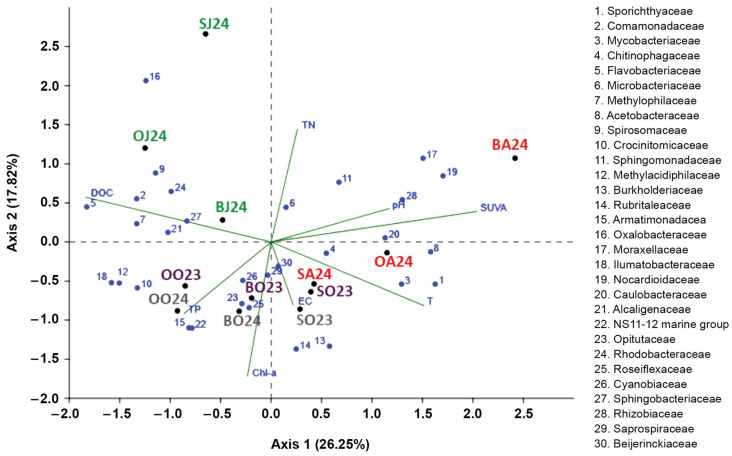
Redundancy Analysis biplot showing the top 30 bacterial families (blue numbers with full names provided in the legend of the figure), environmental variables (green lines) and lake sites during the respective sampling month (coloured labels: Sul (S), Oko (O) and Bub (B) lakes in June 2024 (J24), August 2024 (A24), and October 2023 (O23) and 2024 (O24).

Seasonal similarities among the top 30 bacterial families and the influence of environmental factors on their distribution were revealed by Redundancy Analysis (RDA) (Figure 5). Due to multicollinearity among the nutrient variables, the environmental parameters included in the RDA were pH, EC, temperature, DOC, TN, TP, SUVA_254_ and Chl-a.

The RDA was statistically significant (F = 7.81, perm. *p* = 0.001; 999 permutations) with an adjusted R^2^ of 0.72. Axis 1 (26.25%) reflects a gradient from DOC-rich conditions to lakes with higher pH, SUVA_254_, and temperature, while Axis 2 (17.82%) represents a nutrient–productivity gradient (TN vs. Chl-a). Bacterial families (Flavobacteriaceae, Comamonadaceae, Methylophilaceae, Methylacidiphilaceae, Oxalobacteraceae and Ilumatobacteraceae), positioned on the DOC-aligned side of Axis 1, were associated with terrestrially influenced environments, consistent with taxa adapted to complex organic substrates and low water temperatures. In contrast, families (Sporichthyaceae, Mycobacteriaceae, Acetobacteraceae, Nocardioidaceae, Rhizobiaceae and Caulobacteraceae), occurring on the positive side of Axis 1, displayed positive correlations with higher temperature, pH and SUVA_254_, indicating affiliation with warmer and more alkaline waters enriched in DOC with high aromaticity.

Axis 2 (17.82% of the variance) demonstrated a nutrient productivity gradient. Higher TN levels structured the distribution of families in the upper part of the ordination, including families Microbacteriaceae, Sphingomonadaceae, and Moraxellaceae, whereas families positioned along the lower part of Axis 2 (Mycobacteriaceae, Burkholderiaceae and Rubritaleaceae) were associated with elevated Chl-a, suggesting linkages to phytoplankton-driven resource availability.

Temperature emerged as a major determinant of bacterial community structure. Several families distributed along the temperature vector (primarily in the right-lower quadrant) exhibited strong positive responses to warmer conditions, implying thermal preference or functional traits that support growth in warmer lakes. Conversely, families located opposite the temperature gradient, particularly in DOC-rich or nitrogen-enriched regions of the ordination space, appeared characteristic of cooler and allochthonous organic matter rich waters. This pattern indicates the coexistence of warm-adapted taxa with families specialized for cooler, resource-rich environments.

## 4. Discussion

### 4.1. Lake Environments

The studied environments exhibited typical characteristics of glacial lakes, including relatively low water temperatures, well-oxygenated water, low concentrations of suspended solids, and low nutrient levels, including DOC and DON. The lakes showed low primary productivity and were phosphate-limited, consistent with other glacial ecosystems [11,61,62,63]. Previous reports have shown that, among environmental factors, water temperature is a primary driver of hydrochemical and biological changes in these habitats, activating primary producers, organic matter decomposers, and organic compounds leaching from terrestrial litter [13,61,64]. Based on the water temperature values, the studied lakes were divided into three distinct seasonal clusters corresponding to the sampling period, with mean temperatures of 5.63 ± 1.5 °C (‘cold’), 11.4 ± 0.6 °C (‘transition’), and 18.7 ± 1.9 °C (‘warm’) (Table 1). DOC composition shifted from predominantly recalcitrant carbon (DOC/DON = 49.73) in the ‘cold’ season, to a mixture of recalcitrant and labile carbon (DOC/DON = 15.85) in the ‘transition’ season, and finally to a dominance of labile carbon (DOC/DON = 4.76) in the ‘warm’ season. The ‘cold’ and ‘transition’ clusters corresponded to samples collected in June and October, during which melting snow (June) and autumn rains (October) transported organic matter and nutrients from catchments into the lakes [65,66]. The high Chl-a levels observed in October appear to contradict this pattern. However, the very weak correlation between Chl-a (a proxy of algal biomass) and DOC suggests that algal exudates [67,68] rather than algal biomass primarily contributed to DOC. This observation aligns with previous studies indicating that algal exudation declined to near zero by the end of the growing season [69].

In contrast to the ‘cold’ and ‘transition’ seasons, summer conditions limit terrestrial inflows [70] and promote algal exudation in lakes, making it a primary source of nutrients for bacterial heterotrophs [64,71,72]. In our study, the ‘warm’ season was associated with the lowest DOC and Chl-a concentrations, supporting the hypothesis that increased microbial and grazing activities, likely stimulated by higher water temperatures and the higher lability of algal biomass and exudates, drive this pattern [73,74]. The highest SUVA_254_ value observed during the ‘warm’ season further supports this assumption, indicating greater aromaticity of carbon compounds, probably released by microbial degradation [72,75]. Increased DOC aromaticity may also reflect the increased resistance of algal-derived DOC under phosphorus-limited conditions [76], as indicated by the higher TN/TP ratio observed in this study.

Additionally, water temperature of the lakes showed pronounced altitudinal gradients, evident both in the mean values (8.45 ± 6.5 °C in Sul; 9.76 ± 5.7 °C in Oko; 12.81 ± 5.3 °C in Bub), and the mean daily warming rate (0.29 °C in Sul; 0.23 °C in Oko; 0.19 °C in Bub). Based on these observations, we suggest that water temperature, the rate of temperature change and nutrients (load and quality) influenced bacterial community composition through a ‘species sorting’ mechanism during the monitoring period, thereby favouring taxa best adapted to the prevailing environmental conditions [5,13,77,78].

### 4.2. Seasonal Shifts in Bacterial Diversity

Many authors [11,13,79,80] have linked seasonal variations in microbial communities to fluctuations in environmental factors, identifying temperature as the primary driver, alongside nutrient availability and biological activity. In this study, the highest bacterial richness and diversity were recorded in June (Table 2), coinciding with the lowest water temperatures (Table S1). For example, in June, the number of identified OTUs reached 792, and community diversity, estimated by the Shannon index, averaged 5.59 ± 0.21. As water temperatures increased, both richness and diversity declined, reaching their minimum values in August with only 348 OTUs and an average Shannon index of 4.47 ± 0.36. This summer minimum in bacterial community complexity coincided with water temperatures approximately 13 °C higher than in spring, with Sul Lake experiencing a temperature difference of up to 14.5 °C. The reasons for the substantial OTUs loss—40% in Oko and Bub lakes, and 65% in Sul Lake—remain unclear. However, it can be assumed that August temperatures, potentially exceeding the optimal range for bacterial growth, combined with reduced nutrient availability (DOC and SUVA_254_), inhibitory UV radiation [81], and intensified biological pressures such as grazing and viral infections [82], contributed to this decline. Furthermore, the Venn diagram (Figure 2) showed that: (i) bacterial communities of the colder October 2024 and warmer October 2023 consisted of stable core taxa (16–22%) and much more taxa sorted by actual environmental conditions; and (ii) the total number of shared OTUs per lake increased (38 in Sul, 49 in Oko, and 51 in Bub) with decreasing altitude (2535 m a.s.l. to 2282 m a.s.l.), suggesting that lower temperature fluctuations (coefficient of variation: Sul-77; Oko-58; Bub-46) promote greater between-month (or between-season) community similarity. The most pronounced negative effect on bacterial richness and diversity was observed in Sul Lake, which is situated at the highest elevation and which has the smallest surface area, and warms faster than the other lakes.

The above-mentioned patterns of changes in bacterial richness and diversity suggest that the more ecologically relevant microbial strategy in glacial lakes is to maintain high genetic diversity among niche specialists capable of rapid growth and exploitation of specific environmental resources, rather than relying on a broad-spectrum of generalist taxa.

Additionally, the Venn diagram of OTUs sharing (Figure 2) highlighted the greater complexity and uniqueness of bacterial communities in spring (June), with substantially more unshared OTUs (264 in Sul, 102 in Oko, and 112 in Bub) compared to summer (August), when the number of unshared OTUs sharply declined to 31 in Sul, 37 in Oko, and 41 in Bub. In June, unshared OTUs accounted for 80% of the total in Sul Lake, and 52–54% in Oko and Bub, whereas in August, they represented only 27–35% in each lake. We speculate that the higher community complexity in June results from the interplay of two environmental mechanisms: ‘species sorting’ driven by cold water temperatures and relatively low grazing pressure, and a ‘mass effect’, associated with ice-cover melting and increased terrestrial inflows carrying allochthonous bacteria [13,83].

Overall, bacterial alpha diversity in the Rila Lakes revealed phylogenetically diverse communities, predominantly composed of niche specialists well adapted to cold environments. The observed temporal changes in bacterial community composition support our hypothesis that rising temperatures negatively affect these communities by decreasing diversity and richness, which could, in turn, diminish the intensity and scope of local ecological processes. Sul Lake appears to be the most vulnerable to regional warming, as it exhibited the greatest seasonal (between-month) fluctuations in community structure.

### 4.3. Spatial-Temporal Dynamics in Bacterial Community Composition

#### 4.3.1. Spatial-Temporal Dynamics of Bacterial Phyla

Despite the harsh environmental conditions, the lakes harbour high bacterial diversity, represented by 18 phyla (Figure 3). The core phyla Actinomycetota, Pseudomonadota and Bacteroidota, are among the most frequently reported in freshwater ecosystems worldwide, including glacial lakes [11,12,13,15,61]. Among these, Pseudomonadota was the most diverse group, comprising 269 OTUs, while Actinomycetota was the most abundant, accounting for an average of 47% of the total bacterial abundance. Together, these two phyla represented 72% and 91% of total bacterial abundances during the ‘cold’ and ‘warm’ seasons, respectively. The dominance of Actinomycetota, Pseudomonadota and Bacteroidota in the glacial lakes reflects their high resistance to UV radiation, which contributes to their ability to synthesise pigments [84,85], formation of endospores [86], and high GC content in their DNA [85]. These phyla are also well adapted to oligotrophic environments [9,87], with some capable of rapid reproduction [88] and employing diverse strategies to avoid grazing [89,90]. Moreover, many of them actively contribute to nutrient cycling, especially that of nitrogen (Pseudomonadota, class Alphaproteobacteria) and carbon (Actinomycetota, Bacteroidota, Pseudomonadota) [90,91,92,93].

Although these core phyla persisted throughout the study period in the Rila lakes, distinct seasonal and altitudinal patterns in community composition and structure were observed (Figure 3). Warming of the lakes triggered a process of ‘species sorting’, with the most significant shift observed between the ‘cold’ and ‘transition’ seasons, during which water temperature almost doubled. At that time, OTU richness in both Pseudomonadota and Actinomycetota declined by 42–49%, followed by 12% decrease between the ‘transition’ and ‘warm’ seasons. The number of families followed a similar seasonal decline from 60 → 38 → 32 in Pseudomonadota and from 32 → 25 → 23 in Actinomycetota. This trend of decreasing bacterial richness and diversity suggested that both phyla were predominantly composed of psychrophilic species (niche specialists), which were unable to withstand warming stress and the associated changes in the quantity and quality of the lakes’ nutrients [9].

Additionally, not only a seasonal but also altitudinal gradient in the Actinomycetota shift was observed, and this shift was linked to the warming rate of lake water. The relative abundance of Actinomycetota in bacterial community structure increased more substantially from the ‘cold’ to the ‘warm’ season in Sul Lake (from 38% to 72%), followed by Oko Lake (from 34% to 59%), and Bub Lake (from 50% to 51%). This pattern supports earlier suggestions that colder, high-altitude habitats are more vulnerable to global warming than the low-altitude ecosystems [94], leading to more pronounced shifts in the composition and structure of their bacterial communities.

Overall, these findings indicated that even at the phylum level, significant differences in bacterial community composition and structure occurred along both seasonal and altitudinal temperature gradients. This pattern, along with the observed declines in OTU richness and phylum diversity, supports our hypothesis that rising regional temperatures might negatively impact lake microbiota. Specifically, warming appears to favour the proliferation of phylum Actinomycetota at the expense of overall microbial diversity, potentially influencing key ecological functions within lake ecosystems.

#### 4.3.2. Spatial-Temporal Dynamics of Core Bacterial Families and Genera

The core phyla Actinomycetota, Pseudomonadota and Bacteroidota were represented by 28, 55 and 25 families, respectively, along with numerous OTUs that remained unidentified at family level. Among these, the most abundant families (>5.0% of the total) included Sporichthyaceae and Mycobacteriaceae (Actinomycetota), Comamonadaceae (Pseudomonadota) and Chitinophagaceae (Bacteroidota) (Figure 4). Additionally, several minor taxa, such as Acetobacteraceae, Moraxellaceae, Oxalobacteraceae, Sphingomonadaceae, Burkholderiaceae and Methylophilaceae (Pseudomonadota); Flavobacteriaceae and Crocinitomicaceae (Bacteroidota); Nocardioidaceae (Actinomycetota); Armatimonadaceae (Armatimonadota); and Methylacidiphilaceae (Verrucomicrobiota), showed markedly increased relative abundances during specific seasons.

The core families were widely distributed across the lakes, with an overlap ranging from 44% for Chitinophagaceae to 68% for Sporichthyaceae, and remained consistent across seasons. However, some features of these families were linked to their spatial and temporal dynamics. The dominance of these families in the Rila Lakes aligns with previous findings that identify them as among the most ubiquitous and abundant components of lake bacterioplankton, with a global distribution across diverse limnetic systems characterized by contrasting physical, chemical, and biological conditions [85,90,95,96,97,98]. The widespread occurrence of Sporichthyaceae has been associated with resistance to desiccation, predation, and strong variations in organic carbon loads [99,100,101], while the success of Comamonadaceae is related to their opportunistic life strategies [102].

Within each family, even in the same genus, a consistent pattern of bacterial seasonal shift was observed: a few OTUs persisted across the whole ice-free season, whereas others appeared sporadically in certain months or lakes. Thus, the stability (taxonomic and, likely, metabolic) of bacterial communities in the lakes appears to depend on a limited set of species, with transient enrichment under specific conditions by rapidly proliferating, short-lived phylotypes.

The seasonal dynamics of the core families exhibited distinct patterns in their relative abundances: Sporichthyaceae and Mycobacteriaceae (Actinomycetota) increased from the ‘cold’ to the ‘warm’ season, Comamonadaceae showed a notable decline, while Chitinophagaceae displayed no significant temporal variation. These changes within bacterial communities may result either from shifts in the abundance of the respective taxa or from changes in the abundances of other community members, and most commonly reflect the interplay of both processes. In this study, bacterial transition to the ‘warm’ season was consistently associated with a reduction in OTUs abundances and community diversity to minimal levels, as indicated by the ACE/Chao1 and Shannon diversity indices. OTUs reduction was 25–30% for Sporichthyaceae and Mycobacteriaceae, and 40–43% for Comamonadaceae and Chitinophagaceae. However, the summer OTUs decline in Sporichthyaceae and Mycobacteriaceae was offset by the proliferation of specific genera, including *Mycobacterium* (OTU 1; Mycobacteriaceae), and an unidentified genus (OTU 2) and a member of the *hgcI* clade (OTU 4) of Sporichthyaceae. Their increase during the ‘warm’ season ranged from 78% to 154% compared to the ‘cold’ season.

The observed proliferation of certain genera during summer may be attributed to several factors: (1) ambient temperatures approaching the species’ optimal growth range [85,97,103,104], (2) increased visits of mycobacterial animal hosts to water bodies [105] or (3) reduced dependence on organic carbon for *hgcI* members owing to their light-driven actinorhodopsin-based metabolism [106,107]. RDA confirmed that water temperature positively influences the proliferation of Mycobacteriaceae and Sporichthyaceae (Figure 5).

The relative abundance of Comamonadaceae decreased with increasing water temperature, from the ‘cold’ to the ‘warm’ season. Overall, the dominant genera within this family (*Limnohabitans*, *Polaromonas*, *Paucibacter* and *Rhodoferax*) remained with relatively stable abundance across seasons. In contrast, minor (rare) genera, mostly associated with unidentified OTUs, appeared mainly during the ‘cold’ season, especially in June, and in Sul Lake. It should be noted that the genus *Rhodoferax* is still classified within the family Comamonadaceae in the SILVA database (v138), whereas in GTDB (v214.1, release 08-RS214) it has been reassigned to the family Burkholderiaceae within the phylum Pseudomonadota [108]. The relationship of family relative abundance with DOC concentration indicated that Comamonadaceae rely on DOC [22,109], which is less labile than algal-derived organic matter [110] and declined in the lakes during the ‘warm’ season (Table 1). In this aspect, we assume that Comamonadaceae is more sensitive to increasing water temperature and fluctuations in the bioavailability of nutrients.

In contrast to other core families, the consistent presence of Chitinophagaceae, especially the genera *Ferruginibacter* and *Sediminibacterium*, indicates a high ecological tolerance to environmental fluctuations and likely have a stable role across seasons. *Ferruginibacter* contributes by metabolising complex organic polymers, including extracellular polymeric substances [111], while *Sediminibacterium* appears to occupy the phycosphere, potentially utilizing algal exudates or lysate [112]. Additionally, Peter and Sommaruga [113] linked *Ferruginibacter* abundance to lake water turbidity.

#### 4.3.3. Spatial-Temporal Dynamics of Minor Bacterial Families and Genera

Unlike the abundant families, some ‘minor’ families exhibited short-lived population peaks during specific seasons. Notably, Flavobacteriaceae, Oxalobacteraceae and Burkholderiaceae proliferated during the ‘cold’ season, whereas Acetobacteraceae, Moraxellaceae and Nocardioidaceae peaked during the’ warm’ season. Four of these families (Moraxellaceae, Nocardioidaceae, Acetobacteraceae and Burkholderiaceae) belong to the phylum Pseudomonadota, indicating that this phylum persists in lake waters mainly through niche specialists (with some exceptions in certain Comamonadaceae genera), which rapidly proliferate under certain conditions.

The ‘cold-preferring’ members of Flavobacteriaceae (genus *Flavobacterium*), Oxalobacteraceae (genera [*Aquaspirillum*] arcticum group, *Actimicrobium*, *Massilia* and *Undibacterium*), and Burkholderiaceae (genera *Polynucleobacter* and *Limnobacter*) appeared only in June, particularly in Sul Lake. The abundances of Flavobacteriaceae and Oxalobacteraceae correlated negatively with water temperature and positively with DOC. Members of the family *Burkholderiaceae* (genera *Polynucleobacter* and *Limnobacter*) were also highly abundant in June. However, the RDA indicated a stronger association of this family with Chl-a rather than with water temperature, positioning it in the lower right quadrant of the ordination plot. The contrasting responses of Oxalobacteraceae and Burkholderiaceae, both within the order Burkholderiales, suggest potential competition and niche partitioning based on different carbon substrates. Overall, the seasonal distribution patterns of Flavobacteriaceae, Oxalobacteraceae and Burkholderiaceae reflect their ecological roles as cold-adapted degraders of allochthonous (Flavobacteriaceae and Oxalobacteraceae) and autochthonous (*Burkholderiaceae)* organic matter in freshwater ecosystems [96,113,114].

The ‘warm-preferring’ members of Acetobacteraceae (OTU 9), Moraxellaceae (OTUs 36 and 95) and Nocardioidaceae (OTU 15) were associated with the genera *Rhodovarius*, *Acinetobacter* and *Aeromicrobium*, respectively. These taxa proliferated exclusively in Oko (*Rhodovarius*) and Bub (*Acinetobacter* and *Aeromicrobium*) lakes.

Published data on the presence and ecological roles of *Rhodovarius*, *Acinetobacter* and *Aeromicrobium* in high-mountain environments remain limited. However, given their documented capacities for organic matter degradation [115,116,117], environmental adaptation [118,119,120] and optimal growth temperatures ranging from 25 °C to 37 °C [115,121,122,123], it is plausible that species from these genera contribute to nutrient cycling in warmer aquatic habitats or probably in warm months in glacial lakes.

Overall, clear spatial and temporal patterns in bacterial composition and structure at family level were revealed. The core bacterial families (*Sporichthyaceae*, *Mycobacteriaceae*, *Comamonadaceae* and *Chitinophagaceae*) are widely distributed and consistently present, yet exhibit distinct seasonal shifts driven primarily by changes in water temperature and organic matter characteristics. The warm season is accompanied by strong proliferation of a few temperature-tolerant genera, highlighting the dependence of community stability on a limited set of persistent taxa.

In contrast, minor families show short-lived but pronounced seasonal peaks: cold-preferring *Flavobacteriaceae*, *Oxalobacteraceae* and *Burkholderiaceae* increase early in the ice-free season, while warm-preferring *Acetobacteraceae*, *Moraxellaceae* and *Nocardioidaceae* become abundant in summer. These fluctuations indicate the presence of niche specialists that rapidly respond to temperature changes and varying carbon sources.

## 5. Conclusions

The present study provides a detailed comparison of bacterial communities and specific taxa in three glacial-origin lakes in the Rila Mountains, using next-generation sequencing. The bacterial diversity, encompassing 18 phyla and 165 families, highlights the unique ecological value of these lakes in the region. Most of the recorded taxa, particularly at the phylum level, have also been found in other glacial lakes in the Tibetan Plateau and the Pyrenees. Other taxa, especially at family and genus levels, are specific to the studied object. Clear spatial and temporal shifts in bacterial composition and structure were observed. Bacterial richness and diversity declined from the ‘cold’ to the ‘warm’ season and along the altitudinal gradient from the highest to the lowest situated lake, suggesting negative effects of rising water temperature on bacterial stability and potentially on ecosystem functioning. While a few taxa remained stable during seasonal transitions, most were strongly influenced by changes in water temperature and nutrient availability. Both core and minor taxa contributed to these seasonal shifts in community structure.

Our findings are important for understanding bacterial responses to ongoing regional climate warming, which will continue to exert selective pressure, leading to losses of endemic cold-specialists and stimulating the proliferation of warm-adapted groups. Such microbial reorganisation could have cascading impacts on overall ecosystem functioning and stability. Understanding microbial responses to warming is therefore critical for guiding biodiversity conservation strategies of lakes.

## Figures and Tables

**Table 1:** Characteristics of the lake seasonal clusters with means of water parameters for each of them. Different superscript lowercase letters indicate statistically significant differences (*p* < 0.05, Tukey’s test) among the means of the water parameter in the row.

Parameter	Environmental Clusters		
	Cold	Transition	Warm
T (°C)	5.63 ^a^	11.40 ^b^	18.70 ^c^
pH	7.03 ^a^	7.28 ^ab^	7.46 ^b^
DO (mg/L)	10.05 ^a^	8.87 ^ab^	7.54 ^b^
EC (µS/cm)	25.72 ^a^	24.67 ^a^	36.00 ^a^
DC (mg/L)	5.32 ^a^	6.68 ^a^	1.60 ^b^
DOC (mg/L)	4.39 ^a^	4.55 ^a^	0.34 ^b^
DIC (mg/L)	0.93 ^a^	2.13 ^b^	1.26 ^ab^
DON (mg/L)	0.12 ^a^	0.30 ^b^	0.10 ^a^
TN (mg/L)	2.10 ^a^	2.13 ^a^	2.73 ^a^
TP (mg/L)	0.04 ^a^	0.05 ^b^	0.02 ^c^
Chl-a (µg/L)	3.40 ^a^	1.50 ^a^	1.13 ^a^
TN/TP	70.69 ^a^	39.49 ^a^	132.32 ^b^
DOC/DON	49.73 ^a^	15.85 ^b^	4.76 ^b^
SUVA_254_	0.003 ^a^	0.005 ^a^	0.027 ^b^

**Table 2:** Bacterial abundances (OTUs), richness (Chao1 and ACE indices) and diversity (Shannon and Simpson indices) of water communities from Sul (S), Oko (O) and Bub (B) lakes in June 2024 (J24), August 2024 (A24), and October 2023 (O23) and 2024 (O24).

Sample	OTUs	Shannon	Simpson	Chao1	ACE	Coverage
SO23	133	5.26	0.96	196	220	0.99
OO23	102	4.82	0.92	145	153	0.99
BO23	142	5.69	0.96	216	240	0.98
SJ24	329	5.46	0.93	638	703	0.95
OJ24	194	5.47	0.94	276	278	0.98
BJ24	206	5.83	0.96	298	305	0.97
SA24	114	4.34	0.87	160	165	0.99
OA24	116	4.2	0.88	167	170	0.99
BA24	118	4.88	0.94	171	174	0.98
SO24	113	5.02	0.93	163	167	0.99
OO24	169	6.12	0.97	272	307	0.99
BO24	118	5.34	0.95	172	176	0.99

**Table 3:** Bacterial genera representing the dominant bacterial families, as well as those associated with seasonal peaks in the littoral zones of the Rila Lakes.

Genus	Number of OTUs	Relative Abundance (%)	Genus	Number of OTUs	Relative Abundance (%)
Sporichthyaceae (4 genera)			Moraxellaceae (4 genera)		
hgcI clade	9	49.66	*Acinetobacter*	4	92.01
Cand. *Planktophila*	1	8.21	*Cavicella*	3	5.68
*Sporichthya*	1	0.12	Others (<1.0%)	3	1.80
*Longivirga*	1	0.04	Unidentified	2	0.51
Unidentified	5	41.97	Nocardioidaceae (2 genera)		
Comamonadaceae (13 genera)			*Aeromicrobium*	1	92.82
*Limnohabitans*	4	32.96	*Nocardioides*	4	7.18
*Rhodoferax*	3	19.44	Crocinitomicaceae (1 genera)		
*Paucibacter*	2	19.02	*Fluviicola*	10	100.00
*Polaromonas*	2	11.37	Methylacidiphilaceae (unidentified)		
*Rhizobacter*	4	2.03	Unidentified	2	100.00
Others (<1.0%)	9	2.23	Sphingomonadaceae (10 genera)		
Unidentified	21	12.95	*Sphingorhabdus*	2	34.64
Mycobacteriaceae (1 genera)			*Sphingopyxis*	1	19.56
*Mycobacterium*	5	100.00	*Polymorphobacter*	2	6.83
Flavobacteriaceae (1 genus)			*Sphingomonas*	1	3.60
*Flavobacterium*	19	100.00	*Rhizorhapis*	3	2.04
Chitinophagaceae (7 genera)			Others (<1.0%)	6	1.20
*Ferruginibacter*	11	52.02	Unidentified	4	32.13
*Sediminibacterium*	3	28.30	Armatimonadaceae (1 genera)		
*Parasediminibacterium*	3	9.33	*Armatimonas*	1	100.00
*Dinghuibacter*	2	6.22	Burkholderiaceae (2 genera)		
*Aurantisolimonas*	5	2.77	*Polynucleobacter*	2	98.74
Others (<1.0%)	3	0.12	*Limnobacter*	1	1.26
Unidentified	5	1.24	Oxalobacteraceae (6 genera)		
Acetobacteraceae (5 genera)			[Aquaspirillum] arcticum group	1	31.88
*Rhodovarius*	5	95.82	*Actimicrobium*	1	27.47
*Roseococcus*	1	1.64	*Massilia*	2	16.54
Others (<1.0%)	3	0.50	*Undibacterium*	2	10.51
Unidentified	9	2.04	*Duganella*	1	2.49
			*Noviherbaspirillum*	1	0.36
			Unidentified	5	10.75

## Data Availability

Data available in a publicly accessible repository. The sequencing data have been deposited in the NCBI Sequence Read Archive (SRA) (https://www.ncbi.nlm.nih.gov/sra) under the BioProject accession number PRJNA1291052.

## Supplementary Materials

The following supporting information can be downloaded at: https://www.mdpi.com/article/10.3390/life15121921/s1, Table S1: Water parameter of Rila lakes for Sulzata (Sul), Okoto (Oko) and Bubreka (Bub) in June 2024 (J24), August 2024 (A24), October (O) 2023 (23) and 2024 (24) and (standard deviation); Table S2: Pearson correlation analysis of Rila lakes water parameters (*n* = 36); Table S3: SIMPER analysis of Rila lakes water parameters—overall average dissimilarity of 57%; Table S4: Bacterial families in Sulzata (Sul), Okoto (Oko) and Bubreka (Bub) lakes.

## Abbreviations

The following abbreviations are used in this manuscript:

Bub	Bubreka Lake
Chl-a	Chlorophyll-a
NGS	Next-generation sequencing
T	Water temperature
DIC	Dissolved inorganic carbon
DO	Dissolved oxygen
DOC	Dissolved organic carbon
EC	Electrical conductivity
DON	Dissolved organic nitrogen
Oko	Okoto Lake
OTUs	Operational taxonomic units
RDA	Redundancy analysis
Sul	Sulzata Lake
TN	Total nitrogen
TP	Total phosphorus
SUVA_254_	Specific ultraviolet absorbance at 254 nm
