# Supplementary material for: Bacterial Community Composition and Structure in the Littoral of Rila Mountains Glacial Lakes
Source: Life (Basel). 2025 Dec 15;15(12):1921. doi: 10.3390/life15121921 (PMC12735276; doi:10.3390/life15121921)
Supplement: Supplementary file 1 [file life-15-01921-s001.zip › life-3990977-supplementary.pdf]

Table S1. Water parameter of Rila lakes for Sulzata (Sul), Okoto (Oko) and Bubreka (Bub) in June 2024 (J24), August 2024 (A24), October (O) 2023 (23) and 2024 (24) and (standard deviation).

Sample	T (°C)	pH	DO (mg/L)	DC (mg/L)	DOC (mg/L)	DIC (mg/L)	DON (mg/L)	TN (mg/L)	TP (mg/L)	EC (µS/cm)	Chl-a (µg/L)	$SUVA_{254}$	TN/TP	DOC/DON
**SO23**	7.70<br>(0.5)	6.95<br>(0.14)	8.40<br>(0.1)	5.95<br>(0.009)	5.95	0	0.150<br>(0.06)	1.2<br>(0.59)	0.080<br>(0.04)	19<br>(0.7)	7.99<br>(2.3)	0.002<br>(0.0005)	15.00	39.67
**SJ24**	3.30<br>(1.3)	7.98<br>(0.1)	10.50<br>(0.2)	7.79<br>(0.007)	6.67	1.12<br>(0.135)	0.151<br>(0.033)	2.8<br>(0.55)	0.026<br>(0.011)	13.3<br>(0.4)	0.63<br>(0.42)	0.001<br>(0.0005)	107.69	44.17
**SA24**	17.80<br>(0.2)	7.28<br>(0.1)	7.54<br>(0.4)	3.18<br>(0.032)	0.16	3.02<br>(0.003)	0.162<br>(0.03)	2.0<br>(0.36)	0.023<br>(0.015)	31<br>(5.8)	2.49<br>(0.75)	0.007<br>(0.0005)	86.96	0.99
**SO24**	5.00<br>(0.5)	7.45<br>(0.3)	11.21<br>(0.5)	1.10<br>(0.012)	1.10	0	0.094<br>(0.005)	1.8<br>(0.33)	0.034<br>(0.02)	21<br>(0.5)	6.09<br>(1.6)	0	52.94	11.70
**OO23**	10.70<br>(0.9)	7.01<br>(0.05)	9.10<br>(0.2)	6.97<br>(0.05)	3.62	3.35<br>(0.09)	0.320<br>(0.003)	1.6<br>(0.29)	0.060<br>(0.025)	25<br>(1.6)	0.79<br>(0.34)	0.006<br>(0.0005)	26.67	11.31
**OJ24**	5.90<br>(1.4)	5.80<br>(0.2)	9.70<br>(0.2)	8.76<br>(0.043)	6.81	1.95<br>(0.266)	0.259<br>(0.005)	3.8<br>(0.33)	0.026<br>(0.015)	46<br>(1.0)	0.79<br>(0.45)	0	146.15	26.29
**OA24**	17.40<br>(0.2)	7.36<br>(0.1)	7.13<br>(0.4)	0.87<br>(0.011)	0.29	0.59<br>(0.004)	0.080<br>(0.001)	4.2<br>(0.51)	0.020<br>(0.01)	51<br>(9.6)	0.36<br>(0.22)	0.014<br>(0.0007)	210.00	3.63
**OO24**	5.03<br>(0.1)	7.02<br>(0.1)	11.41<br>(0.3)	5.55<br>(0.041)	3.68	1.87<br>(0.032)	0.071<br>(0.002)	1.6<br>(0.27)	0.033<br>(0.017)	32<br>(0.1)	1.60<br>(0.38)	0.005<br>(0.0005)	48.48	51.83
**BO23**	11.80<br>(0.3)	7.05<br>(0.1)	8.70<br>(0.2)	4.59<br>(0.06)	3.53	1.06<br>(0.08)	0.170<br>(0.002)	1.1<br>(0.31)	0.05<br>(0.023)	24<br>(0.7)	2.76<br>(1.1)	0.010<br>(0.001)	22.00	20.76
**BJ24**	11.70<br>(0.4)	7.77<br>(0.1)	8.80<br>(0.3)	8.49<br>(0.059)	6.50	1.99<br>(0.227)	0.420<br>(0.001)	3.7<br>(0.45)	0.053<br>(0.027)	25<br>(0.5)	0.95<br>(0.64)	0	69.81	15.48
**BA24**	20.90<br>(1.0)	7.75<br>(0.1)	7.94<br>(1.0)	0.75<br>(0.008)	0.57	0.18<br>(0.052)	0.059<br>(0.018)	2.0<br>(0.53)	0.020<br>(0.01)	26<br>(2.3)	0.53<br>(0.27)	0.059<br>(0.002)	100.00	9.66
**BO24**	6.83<br>(0.3)	6.96<br>(0.1)	9.10<br>(0.5)	2.76<br>(0.008)	2.12	0.64<br>(0.005)	0.017<br>(0.001)	1.4<br>(0.34)	0.026<br>(0.012)	23<br>(1.0)	3.28<br>(1.3)	0.008<br>(0.0006)	53.85	124.71

Table S2. Pearson correlation analysis of Rila lakes water parameters (n=36).

	T (°C)	pH	DO (mg/L)	DC (mg/L)	DOC (mg/L)	DIC (mg/L)	DON (mg/L)	TN (mg/L)	TP (mg/L)	EC (µS/cm)	Chl-a (µg/L)	$SUVA_{254}$	TN/TP	DOC/DON
**T (°C)**		0.19	0.00	0.03	0.00	0.05	0.86	0.87	0.30	0.10	0.13	0.00	0.12	0.03
**pH**	0.28		0.73	0.31	0.25	0.14	0.89	0.28	0.64	0.01	0.62	0.27	0.41	0.40
**DO (mg/L)**	-0.84	-0.08		0.15	0.10	0.14	0.83	0.57	0.97	0.13	0.54	0.05	0.19	0.21
**DC (mg/L)**	-0.52	-0.26	0.36		0.00	0.00	0.00	0.04	0.03	0.76	0.10	0.02	0.54	0.69
**DOC (mg/L)**	-0.61	-0.25	0.36	0.96		0.00	0.00	0.07	0.01	0.25	0.90	0.04	0.15	0.49
**DIC (mg/L)**	-0.46	-0.36	0.36	0.86	0.85		0.01	0.05	0.02	0.45	0.04	0.09	0.54	0.49
**DON (mg/L)**	-0.05	-0.04	-0.05	0.74	0.66	0.59		0.01	0.00	0.86	0.25	0.11	0.52	0.14
**TN (mg/L)**	0.03	0.23	-0.13	0.49	0.38	0.46	0.59		0.90	0.15	0.00	0.18	0.01	0.13
**TP (mg/L)**	-0.22	-0.10	-0.01	0.51	0.52	0.54	0.71	-0.03		0.05	0.01	0.06	0.00	0.93
**EC (µS/cm)**	0.34	-0.50	-0.34	-0.08	-0.24	0.19	0.05	0.31	-0.41		0.04	0.96	0.00	0.20
**Chl-a (µg/L)**	-0.32	-0.11	0.14	-0.40	-0.03	-0.48	-0.29	-0.57	0.53	-0.42		0.21	0.00	0.50
**$SUVA_{254}$**	0.70	0.28	-0.46	-0.54	-0.48	-0.42	-0.39	-0.33	-0.46	0.01	-0.31		0.46	0.46
**TN/TP**	0.33	0.18	-0.29	-0.16	-0.31	-0.16	-0.16	0.53	-0.77	0.58	-0.60	0.19		0.18
**DOC/DON**	-0.52	-0.21	0.31	0.10	0.17	0.17	-0.36	-0.37	-0.02	-0.32	0.17	-0.19	-0.33	

Table S3. SIMPER analysis of Rila lakes water parameters – overall average dissimilarity of 57%.

Water parameter	Average dissimilarity	Contribution (%)	Cumulative (%)
DOC/DON	11.69	20.55	20.55
DC (mg/L)	8.66	15.22	35.78
DIC (mg/L)	8.39	14.75	50.53
DON (mg/L)	8.31	14.62	65.15
$SUVA_{254}$	8.30	14.60	79.74
TN (mg/L)	3.44	6.04	85.79
DO (mg/L)	3.37	5.93	91.72
TN/TP	1.99	3.50	95.22
EC (μS/cm)	1.07	1.89	97.10
T (°C)	0.98	1.72	98.82
DOC (mg/L)	0.32	0.57	99.40
Chl-a (μg/L)	0.16	0.28	99.68
TP (mg /L)	0.13	0.22	99.90
pH	0.06	0.09	100

Table S4. Bacterial families in Sulzata (Sul), Okoto (Oko) and Bubreka (Bub) lakes.

Phylum	Family	Sul	Oko	Bub	Phylum	Family	Sul	Oko	Bub
	Acidobacteriaceae (Subgroup 1)	+	-	+	Spirochaetota	Spirochaetaceae	+	-	-
	Solibacteraceae	+	-	-		Chthoniobacteraceae	-	-	+
	Ilumatobacteraceae	+	++	++	Verrucomicrobiota	Terrimicrobiaceae	+	+	+
	Microtrichaceae	-	+	+		Methylacidiphilaceae	-	+	+
	Actinomycetaceae	-	+	-		Opitutaceae	+	+	+
	Corynebacteriaceae	-	+	-		Pedosphaeraceae	+	+	+
	Mycobacteriaceae	+	+	+		Rubritaleaceae	+	+	+
	Nocardiaceae	+	+	-		Verrucomicrobiaceae	+	-	+
	Acidothermaceae	+	+	-		Acetobacteraceae	+	+	+
	Frankiaceae	+	+	-		Inquilinaceae	+	-	-
	Geodermatophilaceae	-	+	+		Caulobacteraceae	+	+	+
	Nakamurellaceae	-	+	-		Hyphomonadaceae	+	+	+
	Sporichthyaceae	++	++	++		Elsteraceae	+	-	-
Acidobacteriota	Kineosporiaceae	+	-	-		Holosporaceae	-	-	+
	Brevibacteriaceae	-	+	-		Micropepsaceae	+	-	-
	Cellulomonadaceae	-	+	-		Paracaedibacteraceae	-	+	+
	Dermabacteraceae	-	+	+		Reyranellaceae	+	+	+
	Intrasporangiaceae	-	+	+		A0839	+	+	+
	Microbacteriaceae	+	+	+	Pseudomonadota	Beijerinckiaceae	+	+	+
	Micromonosporaceae	+	-	+		Devosiaceae	+	-	+
	Nocardioidaceae	+	+	+		Hyphomicrobiaceae	+	-	+
	Propionibacteriaceae	+	+	+		Rhizobiaceae	+	+	+
	Pseudonocardiaceae	+	+	-		Xanthobacteraceae	+	-	+
	Streptomycetaceae	+	+	-		Rhodobacteraceae	+	+	+
	Gaiellaceae	+	+	+		Rhodospirillaceae	-	+	-
	67-14	+	-	+		Fokiniaceae	+	-	+
	Solirubrobacteraceae	+	+	+		Rickettsiaceae	+	-	+
	Armatimonadaceae	+	+	+		SM2D12	+	+	-
Armatimonadota	Chthonomonadaceae	+	-	-		Clade_III	-	-	+
	Fimbriimonadaceae	+	-	+		Sphingomonadaceae	+	+	+
Bacteroidota	Bacteroidaceae	+	-	-		Zavarziniaceae	+	+	+
	Dysgonomonadaceae	-	+	+		A21b	-	+	-
	Paludibacteraceae	+	+	-		Alcaligenaceae	+	+	+
	Prevotellaceae	+	-	-		Burkholderiaceae	+	+	+
	Prolixibacteraceae	+	-	-		Chitinibacteraceae	+	+	+

Bacteroidota	SB-5	-	+	-	Pseudomonadota	Chromobacteriaceae	+	+	+
	Tannerellaceae	+	-	-		Comamonadaceae	++	++	++
	Chitinophagaceae	++	++	++		Gallionellaceae	-	+	-
	Saprospiraceae	-	+	+		Methylophilaceae	+	+	+
	Cytophagaceae	+	+	-		Neisseriaceae	+	-	-
	Hymenobacteraceae	+	+	+		Nitrosomonadaceae	+	+	-
	Microscillaceae	+	+	-		Oxalobacteraceae	+	+	+
	Spirosomaceae	+	+	+		Rhodocyclaceae	+	-	+
	Crocinitomicaceae	+	+	+		SC-I-84	+	-	-
	Flavobacteriaceae	+	+	+		Sulfuricellaceae	+	-	-
	NS9 marine group	-	+	+		Sutterellaceae	+	+	-
	Weeksellaceae	-	+	+		T34	-	+	+
	AKYH767	-	+	+		TRA3-20	-	+	+
	env. OPS 17	+	+	+		Diplorickettsiaceae	+	-	-
	KD3-93	-	+	+		Aeromonadaceae	+	+	-
	LiUU-11-161	+	+	+		Enterobacteriaceae	+	+	+
	NS11-12 marine group	+	+	+		Pasteurellaceae	+	+	+
	Sphingobacteriaceae	++	+	+		Yersiniaceae	+	-	-
Bacillota	Bacillaceae	+	-	+		Legionellaceae	+	+	+
	Planococcaceae	-	+	-		Methylococcaceae	-	+	-
	Erysipelatoclostridiaceae	+	-	-		Methylomonadaceae	+	+	+
	Aerococcaceae	-	+	-		Cellvibrionaceae	+	+	+
	Carnobacteriaceae	+	-	-		Moraxellaceae	+	+	+
	Streptococcaceae	-	+	+		Pseudomonadaceae	+	+	-
	Paenibacillaceae	+	-	+		Solimonadaceae	-	-	+
	Gemellaceae	+	-	+		Steroidobacteraceae	+	+	-
	Staphylococcaceae	+	+	+		Xanthomonadaceae	-	-	+
	Clostridiaceae	+	+	-	Fusobacteriota	Fusobacteriaceae	+	-	+
	Eubacteriaceae	+	-	-	Gemmatimonadota	Gemmatimonadaceae	+	-	+
	Lachnospiraceae	+	-	-	Myxococcota	Myxococcaceae	+	-	-
	Hungateiclostridiaceae	+	-	-		Polyangiaceae	+	+	+
	Ruminococcaceae	+	-	-		Sandaracinaceae	+	-	-
	Family XI	+	-	+	Patescibacteria	LWQ8	+	+	-
	Peptostreptococcaceae	+	-	+		Saccharimonadaceae	+	+	-
	Desulfitobacteriaceae	+	-	-		WWH38	-	+	-
	Veillonellaceae	+	-	+	Planctomycetota	Phycisphaeraceae	-	-	+
Cyanobacteriota	Microcystaceae	+	-	-		Gemmataceae	-	+	+

	Nostocaceae	+	-	+		Isosphaeraceae	-	+	+
	Gloeobacteraceae	-	+	-		Pirellulaceae	-	+	+
	Leptolyngbyaceae	-	+	+	Fibrobacterota	Fibrobacterota	+	+	-
	Nodosilineaceae	-	+	-	Desulfobacterota	Desulfovibrionaceae	+	-	-
	Pseudanabaenaceae	-	+	-		Geobacteraceae	-	-	+
	Cyanobiaceae	+	+	+	Chloroflexota	Chloroflexaceae	-	+	-
	Thermosynechococcaceae	-	-	+		Roseiflexaceae	+	+	+
Bdellovibrionota	Bacteriovoracaceae	+	-	+		JG30-KF-CM45	-	+	+
	Bdellovibrionaceae	-	-	+		Ktedonobacteraceae	+	+	+
	Oligoflexaceae	-	-	+	Dependentiae	UBA12409	+	-	-
						Vermiphilaceae	+	-	-

(+) – detected; (++) detected in high abundance; (-) – not detected
